# Supplementary material for: Small RNA and Transcriptome Sequencing Reveals miRNA Regulation of Floral Thermogenesis in Nelumbo nucifera
Source: Int J Mol Sci. 2020 May 8;21(9):3324. doi: 10.3390/ijms21093324 (PMC7246644; doi:10.3390/ijms21093324)
Supplement: Supplementary file 1 [file ijms-21-03324-s001.zip › Supplementary Files/Supplementary file 1. Table S1.docx]

Table S1

Measurement of receptacle and ambient temperatures at five developmental stages

| Stage | Sample ID | Receptacle temperature(℃) | Air temperature (℃) |
| --- | --- | --- | --- |
| Stage 1 | a-1  a-2  a-3  a-4  a-5 | 28.1  28.0  28.3  28.8  28.5 | 26.7  26.5  26.5  26.8  26.4 |
| Stage 2 | b-1  b-2  b-3  b-4  b-5 | 29.7  31.5  30.6  29.9  29.5 | 26.4  26.2  26.5  26.4  26.3 |
| Stage 3 | c-1  c-2  c-3  c-4  c-5 | 31.4  31.0  31.0  33.2  32.4 | 26.5  26.7  26.8  26.7  26.6 |
| Stage 4 | d-1  d-2  d-3  d-4  d-5 | 30.9  31.0  30.3  30.4  29.4 | 26.2  26.7  26.6  26.6  26.7 |
| Stage 5 | e-1  e-2  e-3  e-4  e-5 | 27.4  27.7  27.5  27.3  27.8 | 26.8  27.0  27.1  27.0  27.2 |

The whole measurement process was carried out from 5 am to 6 am on July 3, 2018.
