# Supplementary material for: Small RNA and Transcriptome Sequencing Reveals miRNA Regulation of Floral Thermogenesis in Nelumbo nucifera
Source: Int J Mol Sci. 2020 May 8;21(9):3324. doi: 10.3390/ijms21093324 (PMC7246644; doi:10.3390/ijms21093324)
Supplement: Supplementary file 1 [file ijms-21-03324-s001.zip › Supplementary Files/Supplementary file 2. Table S2.docx]

**Table S2.** Statistics of small RNA data from 15 libraries.

| **Samples** | **Raw reads** | **Clean reads** | **Q20** | **Mapped reads** |
| --- | --- | --- | --- | --- |
| Stage 1_1 | 29,781,783 | 28,033,774 | 99.20% | 25,133,814(89.66%) |
| Stage 1_2 | 28,791,038 | 27,346,560 | 99.30% | 24,484,091(89.53%) |
| Stage 1_3 | 28,468,813 | 26,523,722 | 99.20% | 23,687,123(89.31%) |
| Stage 2_1 | 29,288,049 | 27,548,594 | 99.20% | 25,013,618(90.80%) |
| Stage 2_2 | 28,354,827 | 27,021,415 | 99.10% | 24,477,402(90.59%) |
| Stage 2_3 | 24,927,440 | 22,963,243 | 99.00% | 20,768,464(90.44%) |
| Stage 3_1 | 29,721,908 | 27,175,056 | 99.30% | 21,602,554(79.49%) |
| Stage 3_2 | 29,843,995 | 27,927,204 | 99.20% | 22,072,361(79.04%) |
| Stage 3_3 | 29,085,538 | 26,852,836 | 99.30% | 21,266,409(79.20%) |
| Stage 4_1 | 30,000,000 | 24,074,429 | 99.20% | 20,786,757(86.34%) |
| Stage 4_2 | 28,937,876 | 23,512,925 | 99.00% | 20,204,457(85.93%) |
| Stage 4_3 | 30,769,230 | 24,142,272 | 99.10% | 20,733,422(85.88%) |
| Stage 5_1 | 29,806,266 | 26,822,782 | 99.10% | 21,516,007(80.22%) |
| Stage 5_2 | 27,364,643 | 23,657,916 | 99.00% | 19,155,663(80.97%) |
| Stage 5_3 | 28,681,091 | 24,479,698 | 99.10% | 19,828,554(81.00%) |
