# Supplementary material for: Small RNA and Transcriptome Sequencing Reveals miRNA Regulation of Floral Thermogenesis in Nelumbo nucifera
Source: Int J Mol Sci. 2020 May 8;21(9):3324. doi: 10.3390/ijms21093324 (PMC7246644; doi:10.3390/ijms21093324)
Supplement: Supplementary file 1 [file ijms-21-03324-s001.zip › Supplementary Files/Supplementary file 5. Table S12.docx]

**Table S12.** Summary of transcriptome data from 15 libraries.

| **Samples** | **Raw reads** | **Clean reads** | **Q20 of Fq1** | **Q20 of Fq2** | **Mapped ratio** |
| --- | --- | --- | --- | --- | --- |
| Stage 1_1 | 127,366,118 | 126,210,332 | 99.01% | 96.55% | 65.36% |
| Stage 1_2 | 130,607,632 | 126,620,672 | 99.37% | 98.22% | 69.18% |
| Stage 1_3 | 130,618,136 | 126,619,886 | 99.45% | 98.42% | 67.74% |
| Stage 2_1 | 128,984,692 | 125,836,884 | 99.44% | 98.35% | 66.37% |
| Stage 2_2 | 130,617,568 | 126,324,178 | 99.46% | 98.48% | 67.83% |
| Stage 2_3 | 129,030,574 | 126,165,640 | 99.40% | 98.34% | 69.23% |
| Stage 3_1 | 130,606,984 | 126,319,706 | 99.47% | 98.44% | 69.46% |
| Stage 3_2 | 130,610,690 | 125,936,890 | 99.51% | 98.56% | 67.50% |
| Stage 3_3 | 130,609,062 | 127,340,280 | 99.47% | 98.08% | 67.59% |
| Stage 4_1 | 130,613,690 | 127,138,946 | 99.39% | 97.72% | 68.76% |
| Stage 4_2 | 130,610,100 | 125,949,118 | 99.55% | 98.55% | 71.06% |
| Stage 4_3 | 130,607,846 | 126,647,466 | 99.43% | 97.75% | 65.66% |
| Stage 5_1 | 130,607,096 | 126,784,988 | 99.47% | 98.49% | 66.21% |
| Stage 5_2 | 131,834,936 | 126,575,834 | 99.22% | 97.22% | 64.22% |
| Stage 5_3 | 123,565,310 | 119,055,696 | 99.49% | 98.67% | 66.40% |
