# Supplementary figures and images for: Small RNA and Transcriptome Sequencing Reveals miRNA Regulation of Floral Thermogenesis in Nelumbo nucifera
Source: Int J Mol Sci. 2020 May 8;21(9):3324. doi: 10.3390/ijms21093324 (PMC7246644; doi:10.3390/ijms21093324)

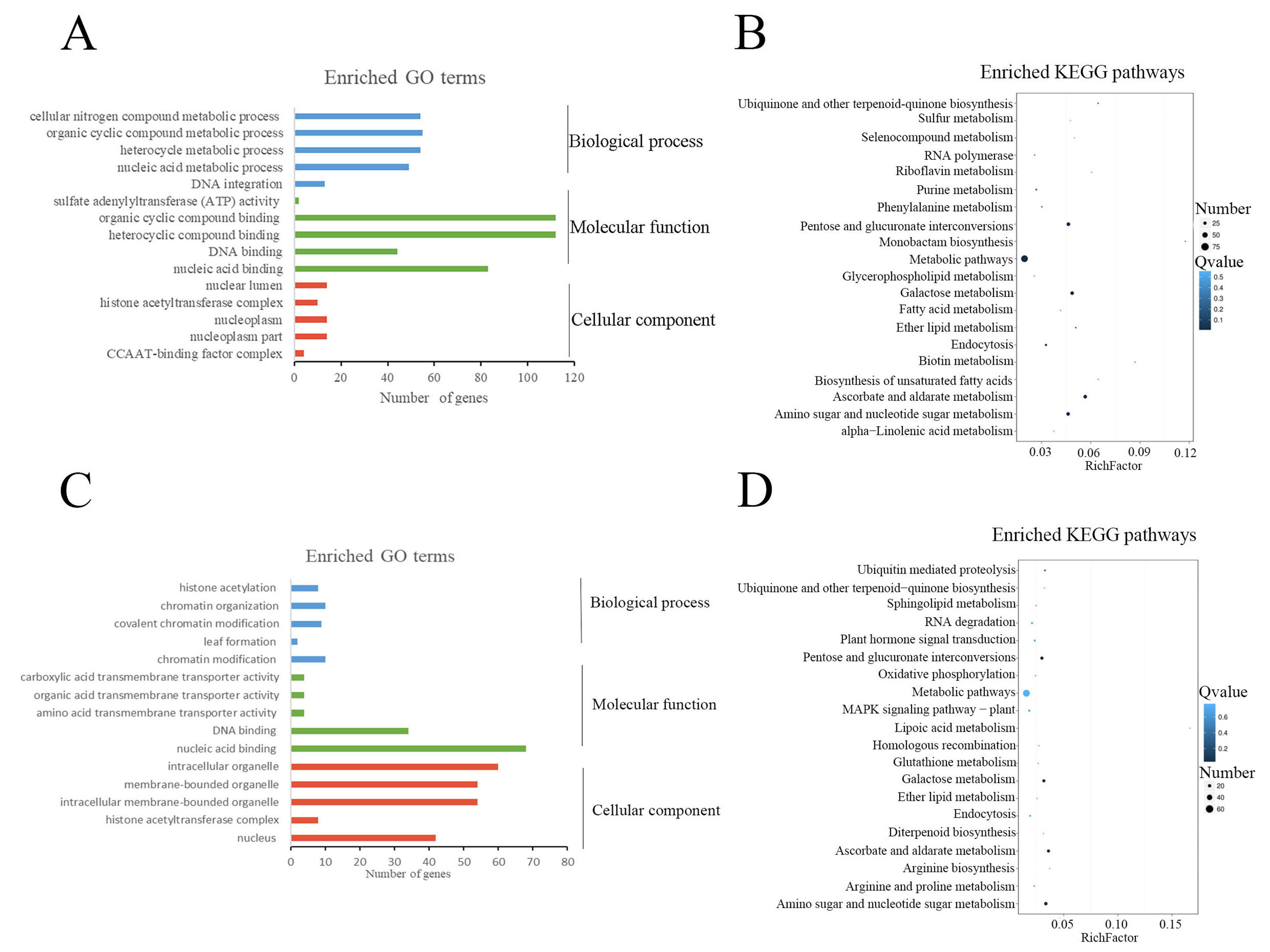

Supplement: Supplementary file 1 [file ijms-21-03324-s001.zip › Supplementary Files/Supplementary file 4/Supplementary file 4. Figure S1.jpg]

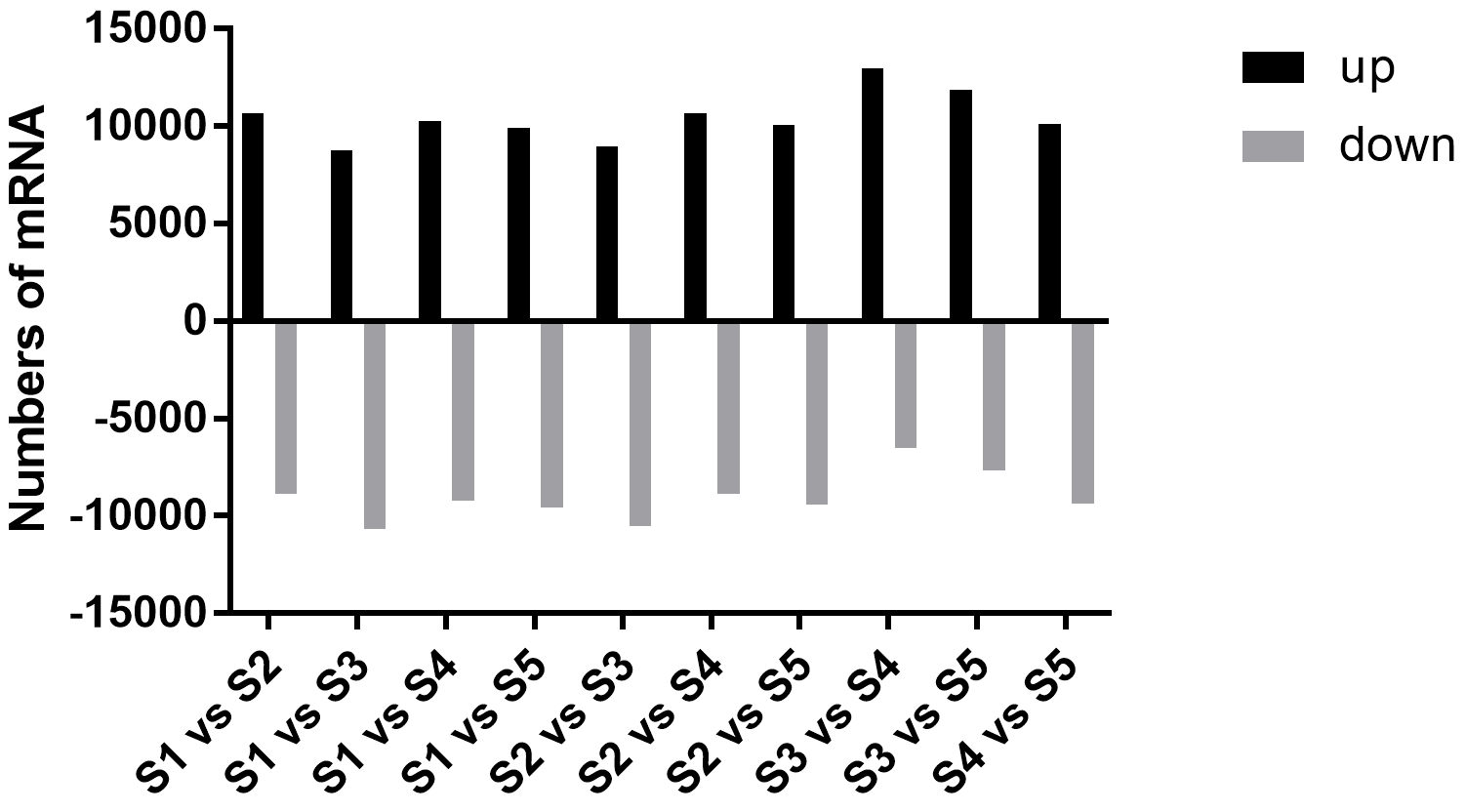

Supplement: Supplementary file 1 [file ijms-21-03324-s001.zip › Supplementary Files/Supplementary file 6. Figure S2.tif]

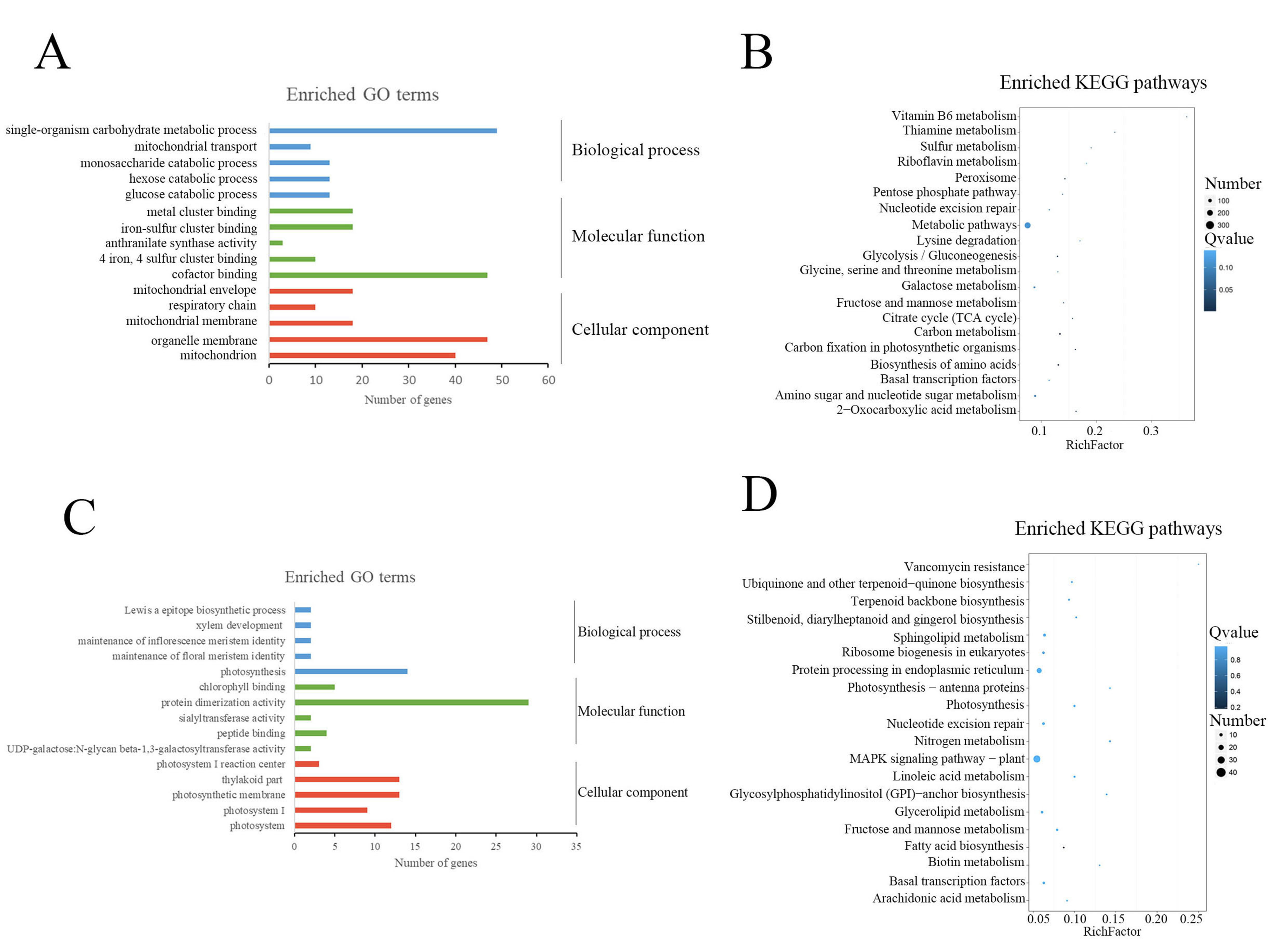

Supplement: Supplementary file 1 [file ijms-21-03324-s001.zip › Supplementary Files/Supplementary file 7/Supplementary file 7. Figure S3.jpg]
